# Diet in Disease

**Published:** 1892-11-26

**Authors:** Ernest Hart

**Affiliations:** Bachelier-ès-Sciences-es-Lettres (rèstreint), formerly Student of the Faculty of Medicine of Paris, and of the London School of Medicine for Women


### Diet in Disease.

By Mrs. Ernest Hart, Bachelier-ès-Sciences-es-Lettres (rèstreint), formerly Student of the Faculty of Medicine of Paris, and of the London School of Medicine for Women.

### VI.—WATER.—SALTS.

**Water.**—When it is remembered that water forms from 60 to 70 per cent. of the body, that it enters into the composition of every living tissue, it can be readily understood how important an article of diet it is. In all the processes of metabolism or constant change by which alone life and function are maintained, water plays a part. Thus, in digestion, the food principle must be dissolved before assimilation can take place; in respiration the expired air is charged with moisture; in circulation the blood must be fluid; in the secretions by means of which the body machine is maintained in working order, and the excretions by means of which the refuse is cast out, the presence of water is in all an absolute necessity.

To drink pure water is the custom of the natural man; to drink impure water is the almost invariable habit of the civilised man. In the days of the Greeks, the Romans, the Egyptians, and the powerful and interesting races of India and Asia Minor; the provision of pure water for the people was looked upon as a national obligation, and the ruins of the great aqueducts by means of which the water of mountain and sky and lake was carried immense distances to the cities, to-day cumber the ground, whence the people now draw their water supply from polluted wells. It is only of recent years, and not till the ravages of cholera and typhoid had at last taught the people and the municipal authorities the lessons which the sanitarians had never ceased to preach in season and out of season; that the absolute necessity of a perfectly pure water supply for drinking purposes was recognised. In London the water supply is derived from the Thames, the Lea, and the New River, rivers which receive sewage and filth of every description during almost the whole length of their course. Elaborate and costly precautions are, however, taken by the water companies to get rid of all the unwholesome and dirty particles with which the water is more or less charged. This is done by filtration and by the formation of large filter beds. Nature's way of cleansing polluted water is twofold—either to pass it through immense filter beds of gravel, under which it collects in underground reservoirs, which may be naturally tapped by "faults" in the strata, or by shafts purposely sunk, which lead to the formation of springs of pure water; or by means of the flow of a river, during which the solid particles sink to the bottom, and the organic pollutions, such as germs, &c., are oxidised and destroyed. Spring or well water often contains inorganic materials dissolved out of the soil through which it has passed, such as lime, iron, &c. River or stream water is the pleasantest to drink, as it contains much air, and a small proportion of organic salts, which give it an agreeable flavour, but so long as it is not considered a crime against humanity and the State to pollute a river or stream, river water is dangerous to drink unfiltered.

**Water is purified in three ways:** by filtration, by distillation, and by boiling.

**Filtration.**—A great variety of filters have been invented. The principle is the same in all of them; to pass the water through some finely granular material, such as gravel, pounded granite, or charcoal, with the intention of arresting the passage of solid particles and of germs. No filter gives absolute security, and in order that it should give even comparative safety it is necessary that it should be kept scrupulously clean. A dirty filter only gives an illusory security.

**Distillation.**—In distillation water is first converted into steam and then cooled again into water. In this way the water is deprived of all its impurities, but also of its entangled air and of its organic salts. Distilled water has, therefore, a flat taste, and in order to make it agreeable it is well to pump carbonic acid gas into it.

**Boiling.**—Water is rendered quite harmless by boiling. In cases where it is not possible to have a pure water supply, where the water can only be obtained from a polluted river or well, which cases are common enough in the county districts in England, and almost invariable abroad, it is more than advisable, it is necessary for the preservation of health, to boil all the water used for drinking purposes. A most useful, and, in fact, indispensable addition to the traveller's luggage when going abroad is a little etna and a spirit lamp. The purchase of a few lemons will enable the traveller then to make a refreshing and inexpensive beverage with water taken from the bedroom caraffe and boiled, without the fear of absorbing at the same time the germs of typhoid fever or cholera. **Boil your water** is the sum and end of all the teachings of the bacteriologists and sanitarians as a protection against infectious diseases carried by water.

**The amount of water** daily required to be taken varies considerably with the individual, some persons losing more by perspiration and the kidneys than others. From 2½ to 4 pints a day is the usual quantity taken, and with most persons much of this is taken in the form of tea, coffee, or beer. A draught of fluid is useful after dinner to wash any undigested particles out of the stomach.

**The Organic Salts.**—The universal custom of eating common salt with food testifies to a need of the body for this particular material. Such indeed is the fact Chloride of sodium or common salt is found in all the tissues and fluids of the body. Without it the blood could not maintain its fluidity, nor could fluids pass through animal membranes. When it is remembered that it is only by the passage of fluids through the membranes of the blood vessels and the lacteals that the products of digestion can be absorbed, it will be seen how important it is to take salt with our food; in fact, if we were entirely deprived of common salt, we should soon die. There are other salts of sodium potassium and magnesium contained in small quantities in the animal and vegetable foods we take, and which are important for the maintenance of the body in health. The salt, however, which is present in the largest amount in the body is lime, as the phosphate of calcium; it forms one-half of our bones, but as the bones when once completely formed, at the age of about 25, do not change in form, the presence of lime in the food is not of such importance in adult life as in youth; in fact, it becomes often rather important to exclude it, as in the case of water highly charged with lime salts. In these cases the lime can be precipitated by boiling.

**Fruit Salts.**—There are a whole class of salts called lactates, lactrates, citrates, malates, and acetates, which are present chiefly in fruits and vegetables. These are converted into carbonates in the body, and they contribute to keep the fluids of the body alkaline, which is necessary so that the functional activity of the body may be carried on. If these salts are not provided, the body is ill-nourished, and finally that condition of mal-nutrition known as scurvy is produced. Though green vegetables have small nutritive value, they always form part of the diet owing to the fact that they furnish the body with the necessary fruit salts. The experience of sailors and Arctic explorers has taught us, often by the severe lesson of terrible suffering, that it is far better to do without the rum ration than without the preserved vegetables, which contain the fruit salt required.

**Condiments** such as pepper, spices, and vinegar give a flavour to food, and perhaps aid digestion by stimulating appetite.

Having considered the various food principles and the part they take in the repair of the body, and its maintenance in health, I will now proceed to describe the processes of digestion by which these various foods are assimilated.

